# Associations between the number of natural teeth and renal dysfunction

**DOI:** 10.1097/MD.0000000000004681

**Published:** 2016-08-26

**Authors:** Hye Min Choi, Kyungdo Han, Yong Gyu Park, Jun-Beom Park

**Affiliations:** a Department of Internal Medicine, Seonam University Myongji Hospital, Goyang, Gyeonggi-do; b Department of Biostatistics; c Department of Periodontics, College of Medicine, The Catholic University of Korea, Seoul, Republic of Korea.

**Keywords:** albuminuria, dentition, epidemiology, glomerula filtration rate, kidney diseases, oral health, tooth loss

## Abstract

The purpose of the present study was to investigate the association between the number of natural teeth and measures of kidney dysfunction, such as urinary albumin/creatinine ratio (ACR) and estimated glomerular filtration (eGFR) rate, using nationally representative data.

The data used were from the Korea National Health and Nutrition Examination Survey with cross-sectional design, which was conducted between 2011 and 2012; the sample analyzed in this study consisted of a total of 10,388 respondents, each of whom was 19 years or older and had no missing outcome variables. The association between the number of natural teeth and kidney function was assessed by multiple logistic regression and model was adjusted for age, sex, waist conference, smoking, drinking, exercise, education, income, frequency of tooth brushing per day, diabetes, metabolic syndrome, urinary ACR, and eGFR.

The mean age, body mass index, and waist circumference were significantly higher among those with lower kidney function (urinary ACR ≥30 mg/g and eGFR <60 mL/min/1.73m^2^). Urinary ACR and eGFR were associated with loss of natural teeth. As urinary ACR increased, the number of natural teeth decreased accordingly. Conversely, the number of natural teeth increased with an increase in eGFR.

This study showed that the number of natural teeth is inversely associated with the presence of kidney disease. Severity of tooth loss may be considered an independent risk indicator for kidney disease among Koreans. More epidemiological studies are warranted to investigate the role of tooth loss in kidney disease, to confirm this relationship and to test possible underlying mechanisms.

## 1 Introduction

Observational studies have shown an association between poor oral health and systemic diseases.^[1,2]^ Periodontitis and pulp necrosis are important sources of systemic microinflammation in chronic kidney disease patients.^[3]^ It was suggested that periodontal disease is associated with increased risk of cardiovascular disease.^[4]^ In the previous study, severe periodontitis was associated with compromised glycemic control and periodontal treatment was associated with improvements in glycemic control in diabetic patients.^[5]^

Chronic kidney diseases were shown to affect the oral health status of patients by inducing xerostomia, delayed eruption of teeth, calcification of root canals, and gingival hyperplasia.^[6]^ The incidence of periodontal disease (bleeding and calculus) was significantly higher among people with renal disease than among controls.^[7]^ Recently, it was reported that periodontitis was associated with increased prevalence of inflammatory markers in end-stage renal disease patients.^[8]^ Recent epidemiological studies have suggested that periodontitis is a major risk factor for renal failure.^[9,10]^ Within the authors’ knowledge, there is no known report to evaluate the association between the number of natural teeth and eGFR with nationally representative data. Thus, this study was performed to investigate the association between the number of natural teeth and both urinary ACR and eGFR using nationally representative data.

## 2 Methods

### 2.1 The design of the study

**Figure 1:**
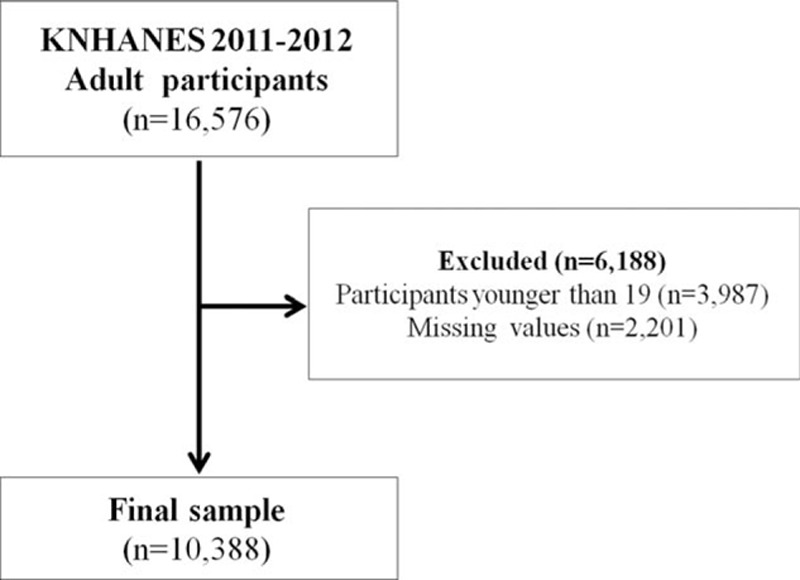
Participant flow chart.

The data from the Korea National Health and Nutrition Examination Survey (KNHANES), which was performed between 2011 and 2012, were used for the study. KNHANES is conducted to evaluate the health and nutritional status for the Korean people and KNHANES is an ongoing surveillance system in the Republic of Korea that assesses the health and nutritional status of Koreans, monitors trends in health risk factors and the prevalence of major chronic diseases, and provides data for the development and evaluation of health policies and programs in Korea. The KNHANES uses a stratified and multistage probability-sampling design with a rolling survey-sampling model. The sample weights were used to calculate all the statistics in this survey. A total of 16,576 patients initially participated in the survey. The analysis in this study was confined to a total of 10,388 respondents who were 19 years or older and who had no missing values for the outcome variables (Fig. 1). This survey was reviewed and approved by the Institutional Review Board of the Korea Centers for Disease Control and Prevention.

### 2.2 Evaluation of kidney function

The level of kidney function is usually determined by urinary albumin/creatinine ratio (ACR) and by estimated glomerular filtration rate (eGFR).^[11,12]^ In this study, urinary ACR and eGFR were used for the categorization and evaluation of the presence of kidney diseases. When urinary ACR was 30 mg/g or greater, the patient was considered to have albuminuria.^[11]^

The level of kidney function was also determined by eGFR using an abbreviated equation: eGFR (mL/min/1.73m^2^) = 186.3 × (serum creatinine^−1.154^) × (age^−0.203^); this result was then multiplied by the constant 0.742 if the patient was female.^[12]^ The equation was developed from the Modification of Diet in Renal Disease (MDRD) formula. Participants with eGFR <60 mL/min/1.73m^2^ were considered to have kidney disease. For this study, chronic kidney disease was ascertained if a patient's eGFR was <60 mL/min/1.73m^2^.^[13,14]^

### 2.3 Anthropometric measurements

Trained staff members performed anthropometric measurements. Height and weight were measured to the nearest 0.1 cm and 0.1 kg, respectively. The participants wore light indoor clothing without shoes during the anthropometric measurements. Waist circumference was measured at the narrowest point between the iliac crest and the lower border of the rib cage.^[15,16]^ Body mass was divided by height squared to calculate body mass index.

### 2.4 Sociodemographic variables

Sociodemographic variables were obtained by the trained interviewers. The self-report questionnaire was used for the smoking status: current smoker or not. Participants were also categorized into 2 groups by the number of drinking episodes (0 vs. 1 or more in the 1-month period before the interview).^[17]^

Participants were regarded as regular physical exercisers if the participants performed vigorous exercise at least 3 times per week for at least 20 minutes per session or moderate exercise at least 5 times per week for at least 30 minutes per session.^[18]^ If the monthly house income was <$1092.40, it was considered the lowest quartile. The participants were categorized by those with at least a high school degree and those with less education. Sleep duration and stress recognition were self-reported. Daily energy, fat, protein, and calcium intake were calculated based on the survey.

### 2.5 Biochemical measurements

Blood pressure measurements were performed using a standard mercury sphygmomanometer (Baumanometer; W.A. Baum Co, Inc, Copiague, NY). Systolic and diastolic blood pressures were measured 2 times at intervals of 5 minutes, and the analysis used the average values.

A blood sample was collected from each participant's antecubital vein after the individual had fasted for >8 hours to measure concentrations of serum-fasting plasma glucose, glycated hemoglobin (HbA1c), total, low-density lipoprotein, high-density lipoprotein cholesterol, triglycerides; white blood cell count, and serum 25-hydroxyvitamin D. The blood samples were analyzed within 24 hours of transportation. Levels of serum-fasting plasma glucose, HbA1c, cholesterol, and triglycerides were measured with the analyzer (Hitachi Automatic Analyzer 7600, Hitachi, Tokyo, Japan) using enzymatic methods and commercially available kits (Daiichi, Tokyo, Japan).^[19]^ A gamma counter (1470 Wizard; PerkinElmer, Wallac, Turku, Finland) and a 25-hydroxyvitamin D ^125^I radioimmunoassay kit (DiaSorin, Stillwater, MN, USA) were utilized to measure serum 25-hydroxyvitamin D levels.

### 2.6 Description of hypertension, diabetes and metabolic syndrome

If participant had a systolic blood pressure of >160 mmHg, a diastolic blood pressure of >90 mmHg, or currently used systemic antihypertensive drugs, the individuals were considered having hypertension.^[20]^ Diabetes was diagnosed when fasting blood sugar was >126 mg/dL or when the individual was currently using antidiabetic medications.^[21]^ Metabolic syndrome was defined according to the criteria for Asians in the American Heart Association/National Heart, Lung, and Blood Institute Scientific Statement.^[22]^ Three or more of the following criteria must be met to be diagnosed with metabolic syndrome: waist circumference of at least 90 cm in men or 80 cm in women; fasting triglycerides of at least 150 mg/dL (or use of lipid-lowering medication); high-density lipoprotein cholesterol of <40 mg/dL in men or 50 mg/dL in women (or use of medication); blood pressure of at least 130 mmHg systolic blood pressure and 85 mmHg diastolic pressure (or use of antihypertensive medication); and fasting blood glucose of at least 100 mg/dL (or use of antidiabetic medication).^[23]^

### 2.7 Oral health behaviors

The time of day of patients’ tooth brushing was noted as part of their oral health behavior and the frequency of daily tooth brushing was calculated from the oral health behavior.

The survey also recorded patients’ secondary oral product use, return dental checkup timing (e.g., within a year), and self-reported oral status.

In this study, the natural tooth was considered to be present if the permanent tooth was present or primary tooth was present. The total number of natural teeth was calculated from the examination. Training was provided to each examiner to minimize measurement errors during the examination as part of quality control.

### 2.8 Statistical analyses

The data were presented as mean ± standard error or as a percent (standard error). To achieve normal distribution, a logarithmic transformation was performed when necessary. Either Student *t* test or a *χ*^2^ test was used to investigate the differences as categorized by urinary ACR or eGFR. The association between the number of natural teeth and kidney function was assessed by multiple logistic regression. Model 1 was adjusted for age, sex, and waist conference. Model 2 was adjusted for the same variables as Model 1 plus smoking, drinking, exercise, education, income, and frequency of tooth brushing per day. Model 3 was adjusted for the variables in Model 2 plus diabetes, metabolic syndrome, urinary ACR, and eGFR. The survey procedure of a statistical program (SAS version 9.2 for Windows, SAS Institute, Cary, NC) was used for statistical analysis to account for the complex sampling design. Two-sided *P* values of 0.05 were used for the statistical significance.

## 3 Results

Table 1 describes baseline characteristics of the study individuals according to kidney function, as categorized by urinary ACR and eGFR. Number of natural teeth was significantly lower among those with lower kidney function (urinary ACR ≥30 mg/g and eGFR <60 mL/min/1.73m^2^). The mean age, body mass index, and waist circumference were significantly higher among those with lower kidney function. Systolic and diastolic blood pressure, glucose, HbA1c, and triglycerides were also statistically higher among those with kidney diseases. High-density lipoprotein levels were significantly lower among those with kidney diseases. The percentage of individuals in the lowest-income quartile was higher among those with lower kidney function. The percentage of patients with at least a high school degree was lower among those with kidney disease. The prevalence of hypertension, diabetes, and metabolic syndrome was significantly higher among those with kidney disease. The subgroup analysis (categorized by number natural teeth for urinary ACR ≥30 mg/g and eGFR <60 mL/min/1.73m^2^) is shown in Table 2. The number of natural teeth was lower among the participants with body mass index of ≥25 (*P* < 0.01). Similarly, the number of natural teeth was lower among the participants with hypertension, diabetes, or metabolic syndrome (*P* < 0.01).

**Table 1:**
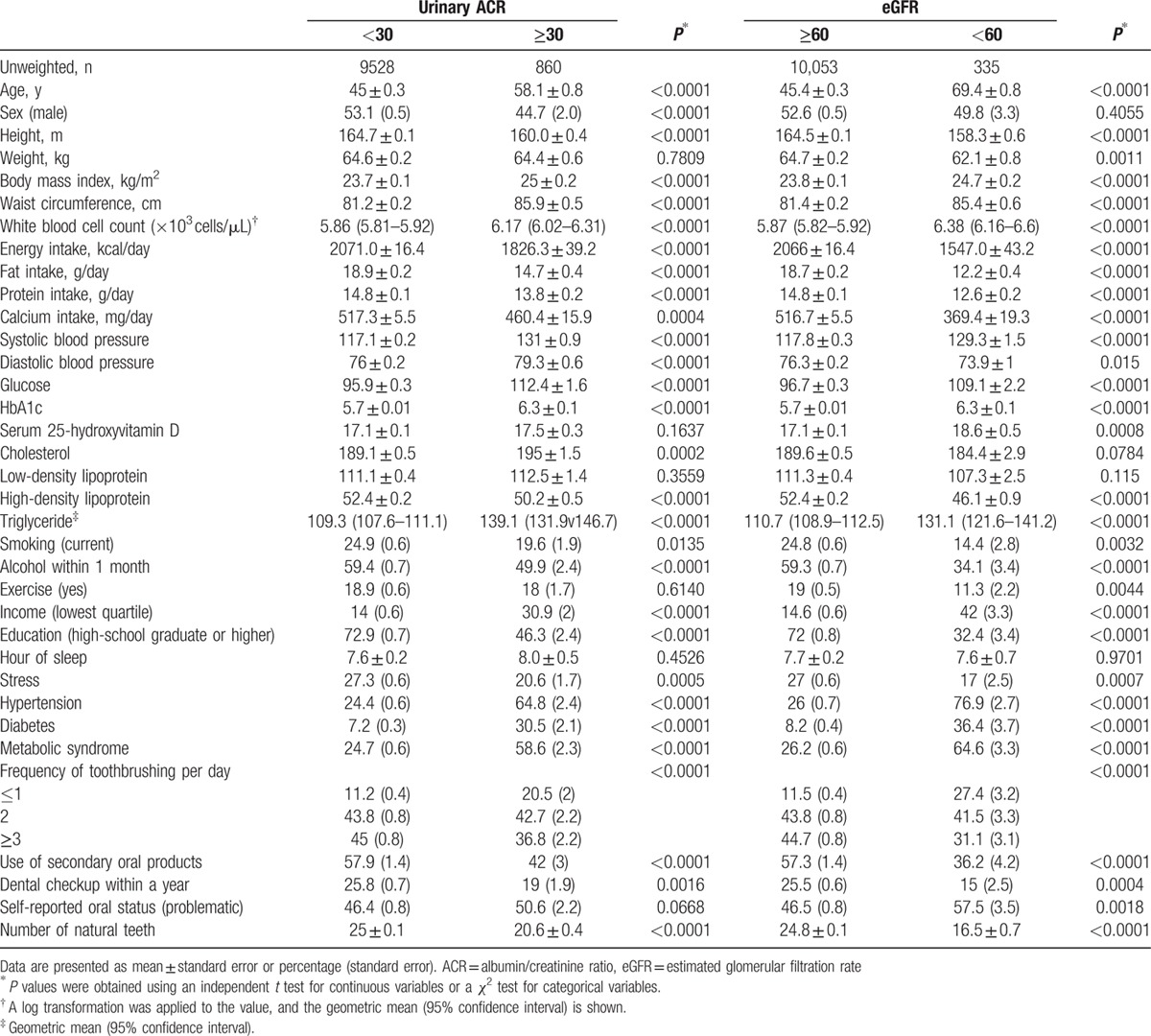
Baseline characteristics of study individuals according to kidney disease, as categorized by urinary ACR and eGFR.

**Table 2:**
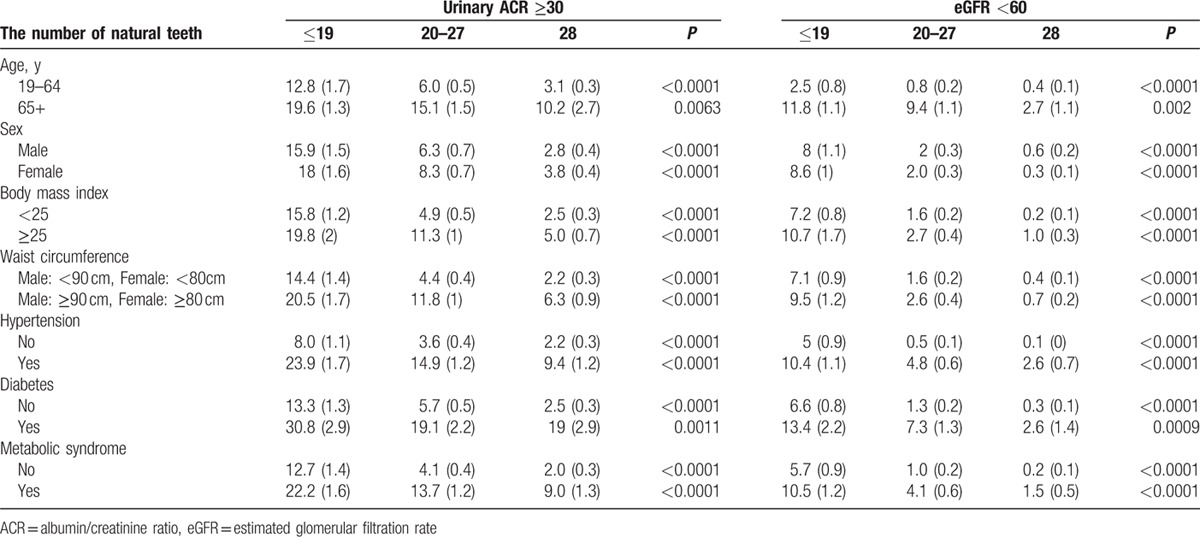
Subgroup analysis for each group in percentage (standard error).

**Figure 2:**
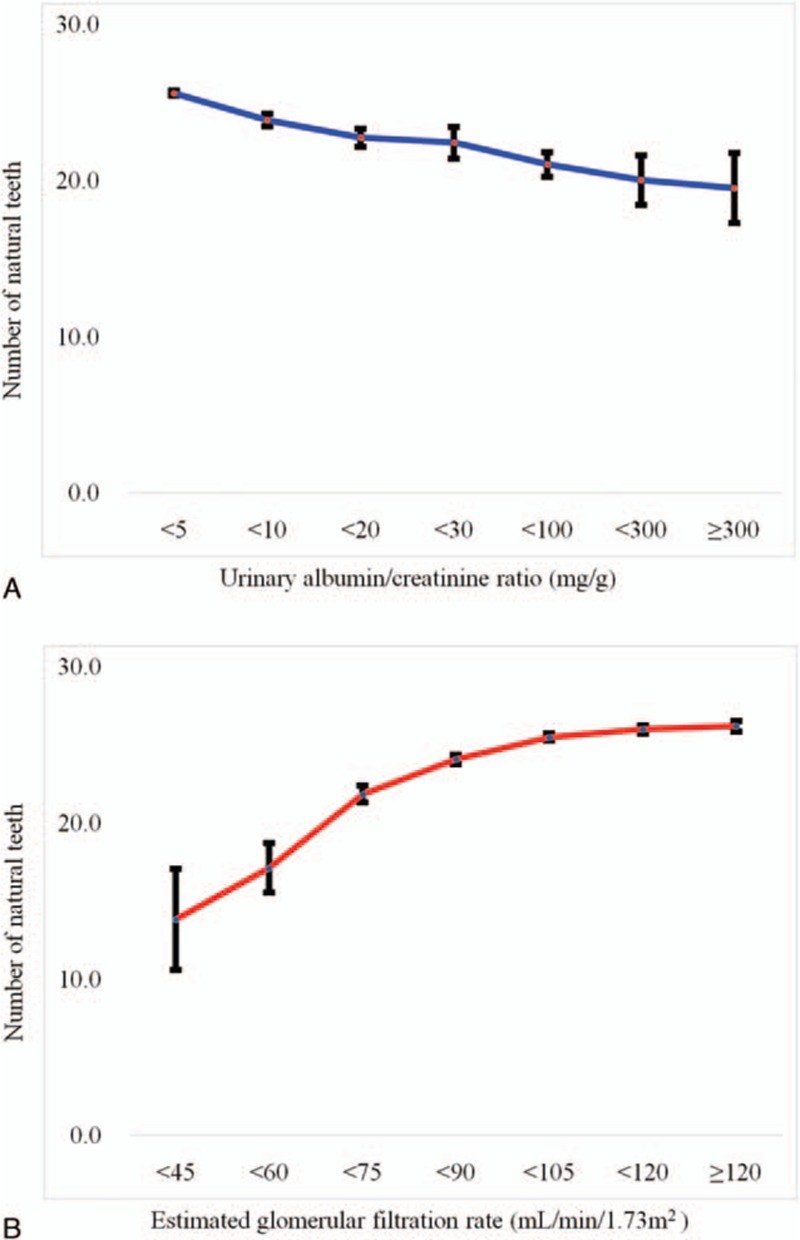
(A) Percentage and standard error for number of natural teeth, as categorized by kidney function using urinary ACR. (B) Percentage and standard error for number of natural teeth, as categorized by kidney function using eGFR.

Figure 2 shows the percentage and standard error for the participants, categorized by number of natural teeth and kidney status. As urinary ACR increased, the number of natural teeth decreased; conversely, the number of natural teeth increased as eGFR increased.

Table 3 shows the adjusted odds ratios and their 95% confidence intervals from the multiple logistic regression analyses for the individuals with kidney diseases (categorized by urinary ACR ≥30 mg/g or eGFR <60 mL/min/1.73m^2^) according to the number of natural teeth. The odds ratios increased in the individuals with urinary ACR ≥30 mg/g. The adjusted odds ratios (with 95% confidence intervals) among participants with urinary ACR ≥30 mg/g were 1 [reference] for individuals with 28 natural teeth, 1.298 (0.989–1.703) for those with 20 to 27 teeth, and 1.758 (1.302–2.375) among those with ≤19 teeth. The adjusted odds ratios (with 95% confidence intervals) for participants with eGFR <60 mL/min/1.73m^2^ were 1 [reference] for individuals with 28 natural teeth, 1.329 (0.691–2.557) for those with 20 to 27 teeth, and 1.287 (0.659–2.513) for those with ≤19 teeth.

**Table 3:**
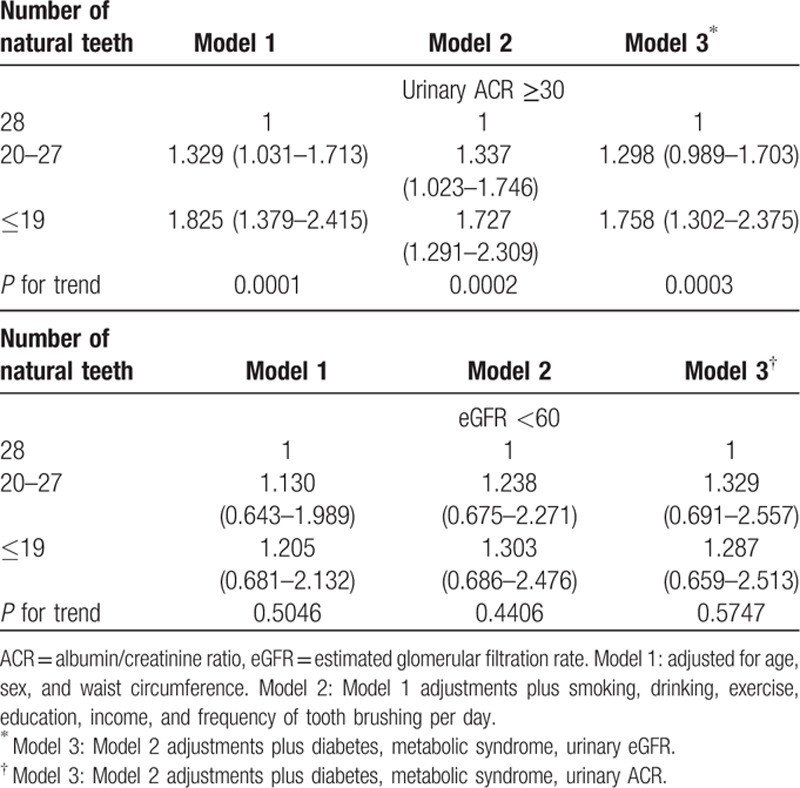
Adjusted odds ratios, 95% confidence intervals, and *P* values in multivariate logistic regression models based on number of natural teeth and kidney status in mean (95% confidence interval).

## 4 Discussion

This study clearly showed that the odd ratios for having fewer natural teeth tended to increase among the participants with lower kidney function (urinary ACR ≥30 mg/g and eGFR <60 mL/min/1.73m^2^).

Kidney function may be determined using various methods.^[12,24–26]^ This study clearly showed that both urinary ACR and eGFR were associated with loss of natural teeth. The urine albumin test is used to evaluate kidney function and to screen people with chronic conditions, such as hypertension and diabetes, which put patients at an increased risk of kidney disease.^[27]^ Albumin is a protein that is present in high concentrations in the blood; it is not usually detected in the urine when the kidneys are functioning properly.^[28]^ Creatinine is a byproduct of muscle metabolism; its level in the urine is usually considered an indication of the urine concentration because it is normally released into the urine at a constant rate.^[29]^ Albuminuria has been shown to be related with periodontal disease.^[30]^ A previous report showed that decreased kidney function (characterized by low eGFR) may be associated with periodontitis.^[24]^ International recommendations suggest that the measurement of serum creatinine should be supplemented with eGFR using the MDRD study's equation.^[31,32]^ This previous report showed that the MDRD equation provided reasonably accurate GFR estimates in patients with chronic kidney disease.^[31]^ The lack of standardization of commercially available creatinine assays, which resulted in varying estimates of GFR, was considered to be problematic, and another study suggested the use of isotope dilution mass spectrometry without the requirement of standardization to the MDRD laboratory.^[32]^ Renal function may be estimated using the Chronic Kidney Disease Epidemiology Collaboration equation.^[25,26]^

The mechanisms behind the association between kidney function and the number of natural teeth may be explained partially by the following. Inflammation can be considered to be the intermediate factor between oral health behavior and systemic diseases.^[33]^ Poor oral health behavior may worsen inflammation; a previous report showed that poor oral hygiene was related to higher inflammatory markers.^[34,35]^ Periodontitis is a chronic inflammatory disease that results from a microbial infection within the subgingival dental plaque biofilm; the resulting inflammatory response may facilitate intravascular dissemination of microorganisms and their products throughout the body.^[36]^ This disease destroys tooth-supporting tissues and may lead to loss of teeth.^[37]^ Periodontitis leading to tooth loss has been labeled as an important potential risk factor for noncommunicable diseases, including diabetes mellitus, cardiovascular diseases, pulmonary diseases, and osteoporosis.^[6]^ A higher percentage of periodontal disease among patients with renal disease was noted when these patients were compared with healthy individuals.^[7]^ The prevalence and severity of chronic periodontitis in the previous cohort of chronic kidney disease patients was markedly higher than it was in a geographically matched control population in Europe.^[38]^ In another report, poor oral health, which includes chronic periodontitis, was a common finding in patients undergoing hemodialysis, and chronic periodontitis was considered a continuous, reversible source of inflammation and this has a potential impact on mortality in patients undergoing hemodialysis.^[39]^ The authors suggested intervention trials to test the hypothesis that treatment of chronic periodontitis may improve survival in patients undergoing hemodialysis.^[39]^

The most important strength of this study is that it is based on a nationally representative sample of Koreans. KNHANES is a nationwide survey of noninstitutionalized civilians; it uses sample participants and sample weights to represent the Korean population and considers survey nonresponse, complex survey design, and poststratification.^[40]^ However, some limitations should be considered regarding this study, as it used a cross-sectional design, which makes it difficult to determine whether there is a direct relationship between the exposure and the outcome.^[41]^ Another limitation of this study is that individuals’ energy intake and oral health behavior were obtained self-reported and were under recall basis.^[42,43]^ Data regarding the inflammatory markers (such as C-reactive protein, tumor necrosis factor-α, and interleukin-6) were not available, so the study's explanation of inflammation and kidney disease is limited.^[44]^

This study showed that the number of natural teeth is inversely associated with the presence of kidney disease. Among those with reduced kidney function (eGFR <60 mL/min/1.73m^2^), the adjusted odds ratios for the group with 28 natural teeth were lower than the ratios for those with fewer natural teeth. Tooth loss may be considered an independent risk indicator for kidney disease in Korean people. More epidemiological studies are warranted to investigate the role of tooth loss in participants with kidney disease, to confirm this relationship, and to test possible underlying mechanisms.

## Acknowledgments

The authors acknowledge the Korea Centers for Disease Control and Prevention for providing the data.

## References

[1] Wilder RS Bell KP Phillips C Dentists’ practice behaviors and perceived barriers regarding oral-systemic evidence: implications for education. *J Dent Educ* 2014; 78:1252–1262. 25179921

[2] Paquette DW Bell KP Phillips C Dentists’ knowledge and opinions of oral-systemic disease relationships: relevance to patient care and education. *J Dent Educ* 2015; 79:626–635. 26034026

[3] Niedzielska I Chudek J Kowol I The odontogenic-related microinflammation in patients with chronic kidney disease. *Ren Fail* 2014; 36:883–888. 24960621 10.3109/0886022X.2014.894764

[4] Madore F Periodontal disease: a modifiable risk factor for cardiovascular disease in ESRD patients? *Kidney Int* 2009; 75:672–674. 19282857 10.1038/ki.2009.15

[5] Casanova L Hughes FJ Preshaw PM Diabetes and periodontal disease: a two-way relationship. *Br Dent J* 2014; 217:433–437. 25342350 10.1038/sj.bdj.2014.907

[6] Wahid A Chaudhry S Ehsan A Bidirectional relationship between chronic kidney disease & periodontal disease. *Pak J Med Sci* 2013; 29:211–215. 24353542 10.12669/pjms.291.2926 PMC3809193

[7] Tiwari V Saxena V Bhambhal A The oral health status of patients with renal disease in central India: a preliminary study. *J Ren Care* 2013; 39:208–213. 24245972 10.1111/j.1755-6686.2013.12040.x

[8] Ariyamuthu VK Nolph KD Ringdahl BE Periodontal disease in chronic kidney disease and end-stage renal disease patients: a review. *Cardiorenal Med* 2013; 3:71–78. 23802000 10.1159/000350046 PMC3678148

[9] Murakami M Suzuki J Yamazaki S High incidence of Aggregatibacter actinomycetemcomitans infection in patients with cerebral infarction and diabetic renal failure: a cross-sectional study. *BMC Infect Dis* 2013; 13:557. 24267704 10.1186/1471-2334-13-557 PMC4222637

[10] Fisher MA Taylor GW Shelton BJ Periodontal disease and other nontraditional risk factors for CKD. *Am J Kidney Dis* 2008; 51:45–52. 18155532 10.1053/j.ajkd.2007.09.018

[11] Han SY Hong JW Noh JH Association of the estimated 24-h urinary sodium excretion with albuminuria in adult koreans: the 2011 Korea National Health and Nutrition Examination Survey. *PloS one* 2014; 9:e109073. 25313865 10.1371/journal.pone.0109073 PMC4196757

[12] Ahn JH Yu JH Ko SH Prevalence and determinants of diabetic nephropathy in Korea: Korea national health and nutrition examination survey. *Diabetes Metab J* 2014; 38:109–119. 24851205 10.4093/dmj.2014.38.2.109 PMC4021298

[13] Park JI Baek H Jung HH CKD and health-related quality of life: The Korea National Health and Nutrition Examination Survey. *Am J Kidney Dis* 2015. 10.1053/j.ajkd.2015.11.005 26706255

[14] So BH Methven S Hair MD Socio-economic status influences chronic kidney disease prevalence in primary care: a community-based cross-sectional analysis. *Nephrol Dial Transplant* 2015; 30:1010–1017. 25586406 10.1093/ndt/gfu408

[15] Weisell RC Body mass index as an indicator of obesity. *Asia Pac J Clin Nutr* 2002; 11 suppl 8:S681–S684.

[16] Oh SW Shin SA Yun YH Cut-off point of BMI and obesity-related comorbidities and mortality in middle-aged Koreans. *Obes Res* 2004; 12:2031–2040. 15687405 10.1038/oby.2004.254

[17] Agarwal DP Cardioprotective effects of light-moderate consumption of alcohol: a review of putative mechanisms. *Alcohol Alcohol* 2002; 37:409–415. 12217928 10.1093/alcalc/37.5.409

[18] Oh JY Yang YJ Kim BS Validity and reliability of Korean version of International Physical Activity Questionnaire (IPAQ) short form. *J Korean Acad Fam Med* 2007; 28:532–541.

[19] Wallace TM Levy JC Matthews DR Use and abuse of HOMA modeling. *Diabetes Care* 2004; 27:1487–1495. 15161807 10.2337/diacare.27.6.1487

[20] Weber MA Julius S Kjeldsen SE Blood pressure dependent and independent effects of antihypertensive treatment on clinical events in the VALUE Trial. *Lancet* 2004; 363:2049–2051. 15207957 10.1016/S0140-6736(04)16456-8

[21] Jeon JY Ko SH Kwon HS Prevalence of diabetes and prediabetes according to fasting plasma glucose and HbA1c. *Diabetes Metab J* 2013; 37:349–357. 24199164 10.4093/dmj.2013.37.5.349 PMC3816136

[22] Chun YH Kim HR Han K Total cholesterol and lipoprotein composition are associated with dry eye disease in Korean women. *Lipids Health Dis* 2013; 12:84. 23734839 10.1186/1476-511X-12-84 PMC3680171

[23] Chin SO Rhee SY Chon S Sarcopenia is independently associated with cardiovascular disease in older Korean adults: the Korea National Health and Nutrition Examination Survey (KNHANES) from 2009. *PLoS One* 2013; 8:e60119. 23533671 10.1371/journal.pone.0060119 PMC3606314

[24] Iwasaki M Taylor GW Sato M Cystatin C-based estimated glomerular filtration rate and periodontitis. *Gerodontology* 2016; 33:328–334. 25294234 10.1111/ger.12159

[25] Choi HY Joo DJ Song MK The power of renal function estimation equations for predicting long-term kidney graft survival: a retrospective comparison of the chronic kidney disease epidemiology collaboration and the modification of diet in renal disease study equations. *Medicine (Baltimore)* 2016; 95:e2682. 26886606 10.1097/MD.0000000000002682 PMC4998606

[26] Terpos E Christoulas D Kastritis E The Chronic Kidney Disease Epidemiology Collaboration cystatin C (CKD-EPI-CysC) equation has an independent prognostic value for overall survival in newly diagnosed patients with symptomatic multiple myeloma; is it time to change from MDRD to CKD-EPI-CysC equations? *Eur J Haematol* 2013; 91:347–355. 23829647 10.1111/ejh.12164

[27] Martinez-Castelao A Gorriz JL Segura-de la Morena J Consensus document for the detection and management of chronic kidney disease. *Nefrologia* 2014; 34:243–262. 24658201 10.3265/Nefrologia.pre2014.Feb.12455

[28] Buser MC Ingber SZ Raines N Urinary and blood cadmium and lead and kidney function: NHANES 2007-2012. *Int J Hyg Environ Health* 2016; 219:261–267. 26852280 10.1016/j.ijheh.2016.01.005 PMC5685486

[29] Ix JH de Boer IH Wassel CL Urinary creatinine excretion rate and mortality in persons with coronary artery disease: the Heart and Soul Study. *Circulation* 2010; 121:1295–1303. 20212276 10.1161/CIRCULATIONAHA.109.924266 PMC2844485

[30] Salimi S Ng N Seliger SL Periodontal disease, renal dysfunction and heightened leukocytosis. *Nephron Clin Pract* 2014; 128:107–114. 25402594 10.1159/000366445

[31] Levey AS Coresh J Greene T Using standardized serum creatinine values in the modification of diet in renal disease study equation for estimating glomerular filtration rate. *Ann Intern Med* 2006; 145:247–254. 16908915 10.7326/0003-4819-145-4-200608150-00004

[32] Vickery S Stevens PE Dalton RN Does the ID-MS traceable MDRD equation work and is it suitable for use with compensated Jaffe and enzymatic creatinine assays? *Nephrol Dial Transplant* 2006; 21:2439–2445. 16720592 10.1093/ndt/gfl249

[33] de Oliveira C Watt R Hamer M Toothbrushing, inflammation, and risk of cardiovascular disease: results from Scottish Health Survey. *BMJ (Clinical research ed)* 2010; 340:c2451. 10.1136/bmj.c2451 PMC2877809 20508025

[34] Akar H Akar GC Carrero JJ Systemic consequences of poor oral health in chronic kidney disease patients. *Clin J Am Soc Nephrol* 2011; 6:218–226. 21115624 10.2215/CJN.05470610

[35] Li X Kolltveit KM Tronstad L Systemic diseases caused by oral infection. *Clin Microbiol Rev* 2000; 13:547–558. 11023956 10.1128/cmr.13.4.547-558.2000 PMC88948

[36] Mohangi GU Singh-Rambirich S Volchansky A Periodontal disease: Mechanisms of infection and inflammation and possible impact on miscellaneous systemic diseases and conditions. *Sadj* 2013; 68:464–467. 24660421

[37] How KY Song KP Chan KG Porphyromonas gingivalis: an overview of periodontopathic pathogen below the gum line. *Front Microbiol* 2016; 7:53. 26903954 10.3389/fmicb.2016.00053 PMC4746253

[38] Sharma P Dietrich T Sidhu A The periodontal health component of the Renal Impairment In Secondary Care (RIISC) cohort study: a description of the rationale, methodology and initial baseline results. *J Clin Periodontol* 2014; 41:653–661. 24738870 10.1111/jcpe.12263

[39] de Souza CM Braosi AP Luczyszyn SM Association among oral health parameters, periodontitis, and its treatment and mortality in patients undergoing hemodialysis. *J Periodontol* 2014; 85:e169–e178. 24224959 10.1902/jop.2013.130427

[40] Han K Ko Y Park YG Associations between the number of natural teeth in postmenopausal women and duration of lactation: The 2010–2012 Korea National Health and Nutrition Examination Survey. *Maturitas* 2016; 85:73–78. 26857883 10.1016/j.maturitas.2015.12.010

[41] Park JB Han K Park YG Association between alcohol consumption and periodontal disease: the 2008 to 2010 Korea National Health and Nutrition Examination Survey. *J Periodontol* 2014; 85:1521–1528. 25008215 10.1902/jop.2014.130782

[42] Han K Hwang E Park JB Association between Consumption of Coffee and the Prevalence of Periodontitis: The 2008–2010 Korea National Health and Nutrition Examination Survey. *PLoS One* 2016; 11:e0158845. 27387296 10.1371/journal.pone.0158845 PMC4936751

[43] Han K Hwang E Park JB Excessive Consumption of Green Tea as a Risk Factor for Periodontal Disease among Korean Adults. *Nutrients* 2016; 8. 10.3390/nu8070408 PMC4963884 27384581

[44] Choi HM Han K Park YG Associations Among Oral Hygiene Behavior and Hypertension Prevalence and Control: The 2008 to 2010 Korea National Health and Nutrition Examination Survey. *J Periodontol* 2015; 86:866–873. 25741579 10.1902/jop.2015.150025

